# Establishment and characterization of a cell line (HCH-1) originating from a human clear cell carcinoma of the ovary

**DOI:** 10.1186/s13048-016-0242-y

**Published:** 2016-06-04

**Authors:** Takashi Yamada, Kimiaki Hattori, Hidetoshi Satomi, Tadashi Okazaki, Hiroshi Mori, Yoshinobu Hirose

**Affiliations:** Department of Pathology, Osaka Medical College, 2-7 Daigaku-machi, Takatsuki, Osaka 569-8686 Japan; Department of Obstetrics and Gynecology, Hirakata City Hospital, 2-14-1 Kin-yahommachi, Hirakata, Osaka 573-1013 Japan; Department of Clinical Pathology, Minoh City Hospital, 5-7-1 Kayano, Minoh, Osaka 562-8562 Japan

**Keywords:** Ovarian clear cell carcinoma, Cell line, MTT assay, CA125, CA19-9, Hot spot, Point mutation

## Abstract

**Background:**

Cell lines are very useful for both clinical and basic research. The establishment of ovarian, malignant tumor cell lines with aggressive histology is especially important. We describe the establishment and characterization of a new human clear cell carcinoma cell line of the ovary.

**Results:**

The cell line HCH-1 was established from an ovarian tumor from a 67-year-old woman. This cell line has grown well for 230 months and has been subcultured more than 50 times. Monolayer cultured cells are polygonal in shape, showing a pavement-like arrangement and a tendency to pile up without contact inhibition. It exhibits a human karyotype with a modal chromosomal number in the hypodiploid range. The cells could be transplanted into the subcutis of SCID mice and produced tumors resembling the original tumor. HCH-1 cells produced CA125 and CA19-9, also identified immunohistochemically in both the original tumor and the heterotransplanted tumors. The cells were sensitive to actinomycin D, carboplatin, cisplatin and mitomycin C, drugs commonly used in the treatment of gynecological cancers. Variant was not found in hotspot of the 50 most commonly reported oncogenes and tumor suppressor genes. Only 12 ovarian clear cell carcinoma cell lines and their characteristics have thus far been reported in the literature. HCH-1 is the first ovarian clear cell carcinoma cell line reported in which the chromosome number is in the hypodiploid range and only the second cell line in which CA125 and CA19-9 are expressed.

**Conclusions:**

Since it is impossible to establish a cell line from the malignant tumor of each patient, the cell line that we established, characterized and report in this paper may be very useful in basic research on ovarian cancer. We have much to learn about the pathogenesis of clear cell carcinoma and this extra line of enquiry may lead us to a better understanding of how to treat and cure this serious disease.

**Electronic supplementary material:**

The online version of this article (doi:10.1186/s13048-016-0242-y) contains supplementary material, which is available to authorized users.

## Background

Clear cell carcinoma of the ovary is an epithelial ovarian cancer that has frequent concurrence with endometriotic lesions [1]. The prognosis is worse than that of any other epithelial ovarian cancer because of its poor chemosensitivity [2]. Previous molecular studies have indicated that mutation in PIK3CA [3], ARID1A [4], and genomic amplification of chr20q13.2 [5] are the most common molecular genetic alterations identified in ovarian clear cell carcinoma. But oncogenesis of ovarian clear cell carcinoma has not been elucidated still now. Therefore, it was considered important to develop an ovarian clear cell carcinoma cell line for clinical and basic research on this disease. We describe here the establishment and characterization of a new human clear cell carcinoma cell line (HCH-1) that expresses CA 125 and CA 19–9.

## Methods

### Materials

**Fig. 1:**
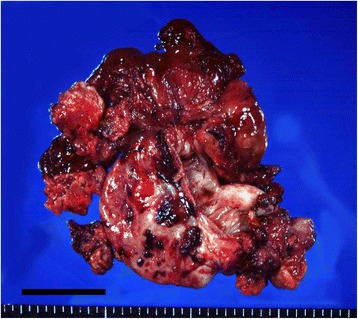
Macroscopic appearance of the original tumor. The right ovarian tumor was cystic with some parts of solid lesions. (bar = 5 cm)

We performed an abdominal simple total hysterectomy, bilateral salpingo-oophorectomy, and omentectomy on a 67-year-old woman with ovarian cancer FIGO stage IIc 19 years ago. The right ovarian tumor was cystic with some parts of solid lesions (Fig. 1). The histology was clear cell carcinoma of the ovary. Preoperative serum tumor markers were CA 125; 340.9 U/ml (normal level <35 U/ml), CA 19–9; 75.4 U/ml (<37 U/ml), α-feto-protein (AFP); 8.2 ng/ml (<20 ng/ml), carcinoembryonic antigen (CEA); 1.3 ng/ml (<5.0 ng/ml), and squamous cell carcinoma (SCC) antigen; 0.6 ng/ml (<1.5 ng/ml). The patient was treated with intraperitoneal 100 mg cisplatin and 400 mg etoposide twice, and intravenous 450 mg carboplatin, 60 mg pirarubicin hydrochloride, 400 mg cyclophosphamide four times. However her clinical condition deteriorated progressively because of the brain metastasis with increasing CA 125. Although the metastatic tumor was resected by surgery, showing adenocarcinoma, she died 23 months after the first surgery. Unfortunately we had not an opportunity to culture the metastatic tumor. The patient gave written informed consent before surgery according to institutional guideline.

### Culture techniques and culture media

The material from the ovarian tumor was finely minced with a pair of sharp blades in a dish including serum-free Ham’s F-12 medium (Flow Laboratories Inc., McLean, VA, USA), stirred slowly with a magnetic stirrer in a 0.25 % trypsin solution (Flow Laboratories Inc.), centrifuged at 70 g for 5 min and placed in culture medium at 37 °C in humidified 5 % CO_2_ and 95 % air. Cells were cultured in Ham’s F-12 medium plus 20 % precolostrum newborn calf serum (Mitsubishi Chemical Industries Ltd., Tokyo, Japan) with kanamycin. Then subcultures were made with 0.1 % trypsin and 0.02 % ethylenediamine-tetraacetic acid (EDTA) solution every 4 weeks. Three months after primary culture the concentration of precolostrum newborn calf serum in the culture medium was reduced from 20 to 10 %.

### Morphology of the culture material and cultured cells

Living cells grown in culture flasks were examined using a phase contrast microscope. For light microscopy, the original tumor was fixed in 10 % formalin, embedded in paraffin, and 4 μm-sections were stained with hematoxylin-eosin (HE). Monolayer cells cultured on slides were fixed in 90 % ethanol and stained by Papanicolaou’s procedure [6].

### Growth characteristics

Cell characteristics were examined chronologically. Suspensions of 1×10^5^ cells each were placed on 35 mm plastic dishes. Cells were incubated for 15 days, and cells in two dishes were counted by an automatic cell counter (Coulter Counter^R^, Coulter Electronics, Luton, England) every other day. The population doubling time and saturation density were calculated from the growth curve. For studies of plating efficiency, 1×10^2^ and 2×10^2^ single-suspension cells were placed onto five-60 mm plastic dishes each and cultured for 14 days. Plating efficiency was determined by the ratio of the number of colonies (more than 10 cells) to the total number of inoculated cells. For the mitotic index, the monolayer cells were cultured for 5 days and treated with 1×10^−7^ M colcemid (Demecolcine Solution, Wako Pure Chemical Industries, Osaka, Japan) for 4 h, placed in a 0.2 % KCl solution for 15 min, and then fixed step-by-step in a methanol:acetic acid solution (3:1). After air-drying, the cells were stained with Giemsa and the mitoses in 1000 cells were counted.

### Chromosome analysis

Histograms of chromosome number distribution were determined on 50 metaphase plates. Their karyotypes were analyzed in 9 cells in accordance with the International System for Human Cytogenetic Nomenclature (ISCN 1995).

### Heterotransplantation

Ten million cells (passage 10) were inoculated subcutaneously into the backs of 6-week-old SCID mice (SCID/Os; Shionogi, Osaka, Japan). When the tumors had grown to 5–10 mm in diameter after 4 weeks, they were removed and processed for morphological examinations. For light microscopy, the excised tumors were fixed in 10 % formalin, embedded in paraffin, and stained with HE. For electron microscopy, the original tumor was fixed by immersion in a mixture of 1.25 % glutaraldehyde and 1 % paraformaldehyde buffered with phosphate saline, pH 7.4, at 4 °C for 3 h. After washing with phosphate buffered saline (PBS), the tumor was postfixed with 1 % osmium tetroxide at 4 °C for 1 h. The tumor was then washed in PBS, dehydrated in graded concentrations of ethanol, and embedded in Epon 812. Sections 0.5 μm thick were cut with a 6000 ultramicrotome (Sorvall, Du Pont, CT, USA) and stained with toluidine blue. Ultrathin sections exhibiting a light gold interference color were cut from appropriate areas chosen from the toluidine blue stained sections, double stained with uranyl acetate and lead citrate, and observed under a JEM-100SX electron microscope (JEOL, Tokyo, Japan) at 80 kV [7].

### Tumor markers

Culture medium in which 2×10^6^ cells/5 ml were cultured for 7 days was examined for AFP, CA 125, CA 19–9, CEA, human chorionic gonadotropin (HCG), SCC antigen, and tissue polypeptide antigen (TPA) by radioimmunoassay or chemiluminescent immunoassay.

### Immunohistochemical stainings

The deparaffinized sections of the original tumor and heterotransplanted tumor on glass slides were stained immunohistochemically using the universal Immuno-enzyme Polymer (UIP) method (HISTOFINE simple stain MAX PO; Nichirei Bioscience Inc., Tokyo, Japan). They were immersed in 0.03 % hydrogen peroxidase and absolute methanol to block endogenous peroxidase. The samples were then washed with phosphate buffered saline and heated in a microwave oven for 10 min at a power of 700 W. After cooling, the slides were incubated with a primary antibody at 4 °C for 12 h and then added and reacted with Histfine Simple stain MAX PO at room temperature for 30 min, followed by diaminobenzidine (DAB) for sections for 5–10 min. Antibodies used as tumor markers were CA 125 (DAKO, Glostrup, Denmark), CA 19–9 (DAKO) and, as hormone receptor markers, were anti-human estrogen receptor α (Nichirei) and anti-human progesterone receptor (Nichirei). Hepatocyte factor-1β (HNF-1β) [8] (Santa Cruz Biotechnology, Inc., Santa Cruz, CA, USA) and Annexin IV [9] (Santa Cruz Biotech) which commonly express in ovarian clear cell carcinoma were also used.

### Chemosensitivity assays

A comparison was made between the effects of actinomycin D (ACD, MSD KK, Tokyo, Japan), doxorubicin (ADM, Kyowa Hakko Kirin Co., Ltd., Tokyo, Japan), 5-fluorouracil (5-FU, Kyowa Hakko Kirin Co.), mitomycin C (MMC, Kyowa Hakko Kirin Co.), bleomycin (BLM, Nippon Kayaku Co., Ltd., Tokyo, Japan), vinblastine (VLB, Nippon Kayaku Co.), vincristine (VCR, Nippon Kayaku Co.), carboplatin (CBDCA, Bristol-Myers K.K., Tokyo, Japan), cisplatin (CDDP, Bristol-Myers), etoposide (VP-16, Bristol-Myers), paclitaxel (PTX, Bristol-Myers) [10], 4-hydroperoxy-cyclophosphamide (CPA, Shionogi & Co., Ltd., Osaka, Japan), irinotecan SN-38 (CPT-11, Yakult Honsha Co., Ltd., Tokyo, Japan) [11] and methotrexate (MTX, Pfizer Japan Inc., Tokyo, Japan) which are often used to treat gynecological cancers [12]. They were dissolved in culture medium and used immediately. For 3-(4,5-dimethyl-2-thiazolyl)-2,5-diphenyl-2H tetrazolium bromide (MTT) assay, 96-well micro well plates were set up in quadruplicate, each well containing 5×10^3^ cells in 100 μl medium. For continuous drug exposure experiments, various diluted drugs in 50 μl were added after 48 h of incubation. The wells were incubated for 72 h after the addition of drugs. MTT (50 μl of 2 mg/ml; Wako Pure Chemical Industries) was added to each well, and the plates were incubated for 4 h. The medium was then removed, 150 μl of dimethyl sulfoxide (DMSO, Sigma, St. Louis, MO, USA) was added to each well, and the plates were agitated for 5 min. The optical density was then read at 570 nm on a microplate reader (Bio-Rad Laboratories, Hercules, CA, USA) and the effective concentration for 50 % kill was calculated from the dose response curve (EC50: dose of drug required to reduce final cell number or optical density in MTT assay to 50 % of control) [13].

### Mutational analysis

DNA was extracted from cultured cells using DNA extraction Kit (PureLink™ Genomic DNA Mini Kit, Invitrogen, Life Technologies, Carlsbad, CA, USA). Somatic mutations (substitutions, insertions or deletions) were assessed using the Ion AmpliSeq™ Cancer Hotspot Panel v2, designed to amplify 207 amplicons covering ~2800 COSMIC mutations from the 50 most commonly reported oncogenes and tumor suppressor genes: ABL1, AKT1, ALK, APC, ATM, BRAF, CDH1, CDKN2A, CSF1R, CTNNB1, EGFR, ERBB2, ERBB4, EZH2, FBXW7, FGFR1, FGFR2, FGFR3, FLT3, GNA11, GNAQ, GNAS, HNF1A, HRAS, IDH1, IDH2, JAK2, JAK3, KDR, KIT, KRAS, MET, MLH1, MPL, NOTCH1, NPM1, NRAS, PDGFRA, PIK3CA, PTEN, PTPN11, RB1, RET, SMAD4, SMARCB1, SMO, SRC, STK11, TP53 and VHL (Ion Torrent, Life Technologies) [14, 15] (Additional file 1: Table S1).

## Results

### Histopathology of the culture specimen

**Fig. 2:**
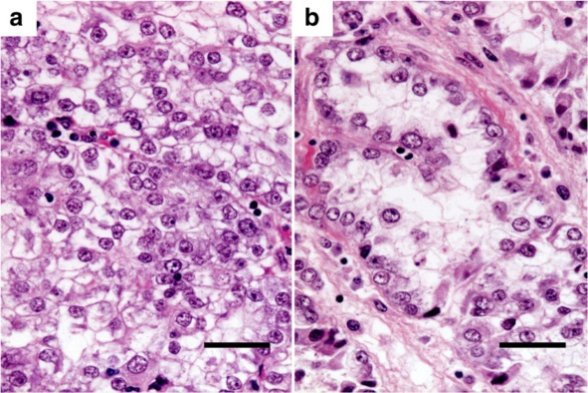
Histology of the original tumor. The right ovarian tumor shows clear cell carcinoma, with a clear cytoplasm (**a**) and a partial hobnail appearance (**b**), stained with hematoxylin and eosin (HE). (bar = 50 μm)

Light microscopy revealed the original tumor to be clear cell carcinoma, with a clear cytoplasm (Fig. 2a) and a partial hobnail appearance (Fig. 2b).

### Establishment of the cell line

After a 2-month stationary period, the fragments developed distinct out-growths. Initially, contamination of spindle shaped fibroblasts was observed, but they disappeared from the cultures upon passaging the cells. The HCH-1 cells grew well, without interruption, for more than 230 months, and more than 50 serial passages were successively carried out. They continue to exhibit stable growth.

### Morphology of the cultured cells

**Fig. 3:**
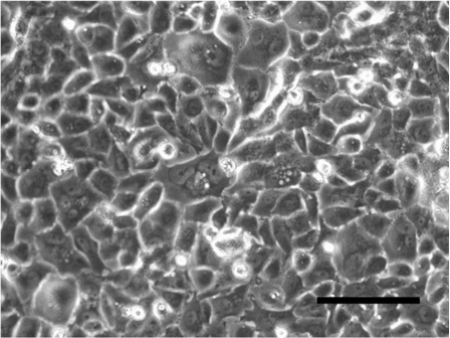
Phase contrast microscopy. It reveals a pavement-like arrangement. (bar = 100 μm)

**Fig. 4:**
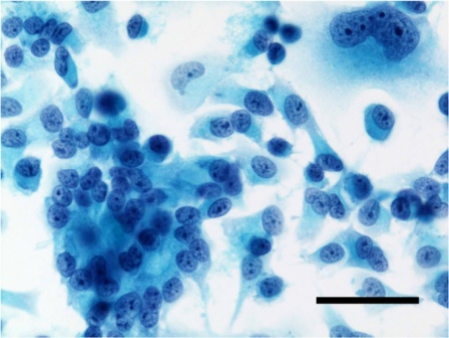
Cytopathology of cultured HCH-1 cells. Multinucleated giant cells with spindle-shaped or multipolar cytoplasm were observed with Papanicolaou stain. (bar = 50 μm)

The monolayer cultured cells appeared to be epithelial, showing a pavement-like arrangement. Multilayering was observed and confluency could not be achieved (Fig. 3). The cells were polygonal and showed such neoplastic features as bizarre aggregation of chromatin granules, thickened nuclear membrane, and multiple large nucleoli. Multinucleated giant cells were also seen (Fig. 4).

### Growth characteristics

**Fig. 5:**
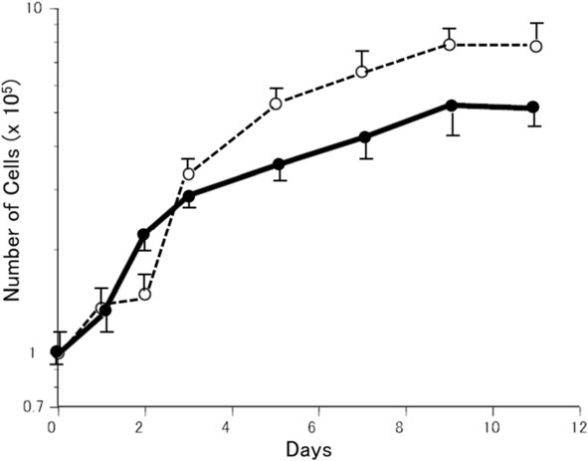
Growth curves of HCH-1. (the 6th(●) and 25th(○) generations.)

The growth curve was examined in passages 6 and 25 of the HCH-1 cell line. Three days after culturing, the cells grew logarithmically (Fig. 5). The population doubling time, the saturation density, the plating efficiency and the mitotic index were 66.4 h, 5.4×10^4^ cells/cm^2^, 18.8 %, 2.7 % in the 6th generation and 48.7 h, 8.2×10^4^ cells/cm^2^, 12.9 %, 8.5 % in the 25th generation respectively.

### Chromosomes

**Fig. 6:**
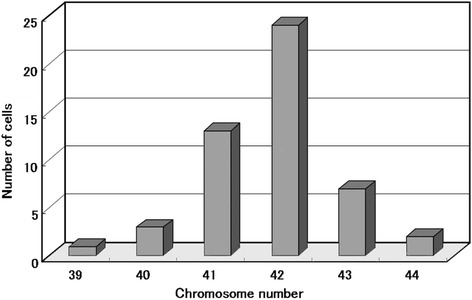
Distribution of chromosomal numbers in HCH-1 (6th generation). The modal number is in the hypo-diploid range

**Fig. 7:**
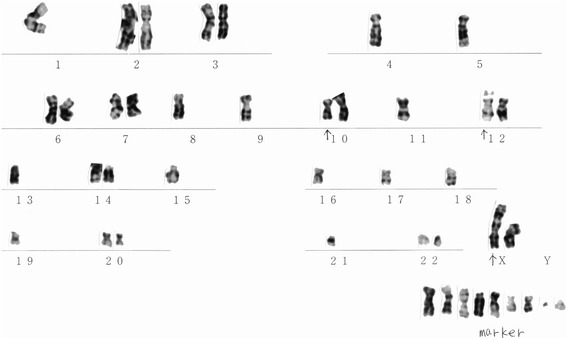
Karyotype of HCH-1 (6th generation). Chromosomal analysis showed various abnormalities

A modal chromosome number was in the hypodiploid range (39–44) (Fig. 6). Chromosomal analysis showed the following abnormalities; 40–42, X, der(X)t(X;1)(p22;q21-23) [7], −1 [9], −4 [9], −5 [7], −8 [3], −9 [6], −10 [3], −11 [9],-12 [3], add (12)(p13) [7], −13 [9], −15 [9], −16 [9], −17 [5], −18 [7], add (18)(q23) [3], +19 [2], −19 [4], +20 [3], −21 [4], −22 [3], +5-9mar (Fig. 7).

### Heterotransplantation

**Fig. 8:**
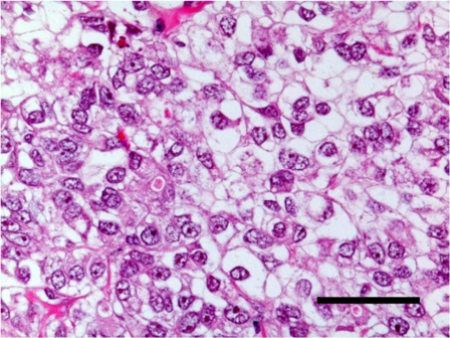
Micrograph of a tumor transplanted into a SCID mouse. It shows clear cell carcinoma closely resembling the original tumor. (HE, bar = 50 μm)

**Fig. 9:**
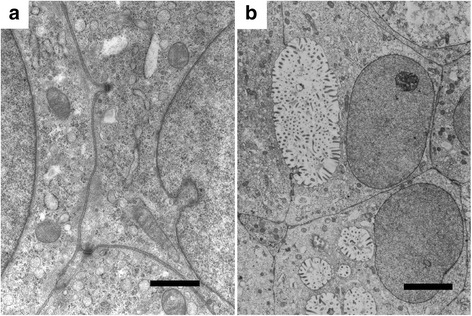
Electron micrograph of a tumor transplanted into a SCID mouse. The cells were attached via desmosome junctions (**a**) (bar = 4 μm), had indented nuclei in the cytoplasm and numerous microvilli on the surfaces (**b**) (bar = 1 μm)

Histopathologically, the transplanted tumors were clear cell carcinoma i.e. with clear cytoplasm, closely resembling the original tumor (Fig. 8). Electron microscopy revealed that adjacent cells possessed desmosome-like junctions (Fig. 9a). The cells had indented nuclei and abundant mitochondria in the cytoplasm, and numerous microvilli on the surfaces (Fig. 9b). These features suggest that the cells were epithelial in origin.

### Tumor markers

Positive levels were observed for: CA 125, 1400 U/ml; CA 19–9, 355 U/ml; and TPA, >1500 U/L. Negative markers were: AFP, 1 ng/ml; CEA, 0.7 ng/ml; CA 72–4, 8.8 U/ml; HCG, <1 mIU/ml; and SCC antigen, <0.5 ng/ml.

### Immunohistochemical stainings

**Fig. 10:**
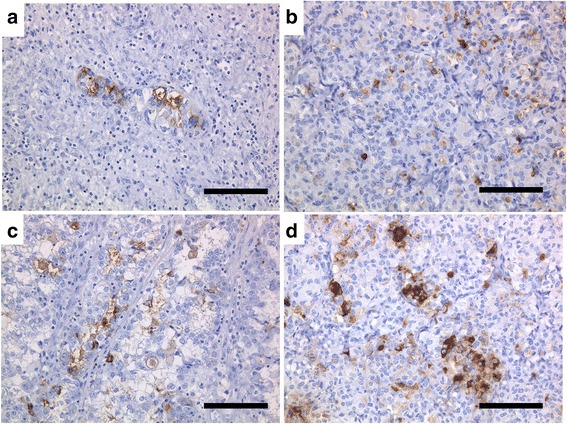
Immunohistochemical stainings of the original tumor (**a**, **c**) and heterotransplanted tumor (**b**, **d**) with CA 125 (**a**, **b**) and CA 19–9 (**c**, **d**). Cancer cells were positive for CA 125 and CA 19–9 (**a**, **b**, **c**, **d**; bar = 100 μm)

**Fig. 11:**
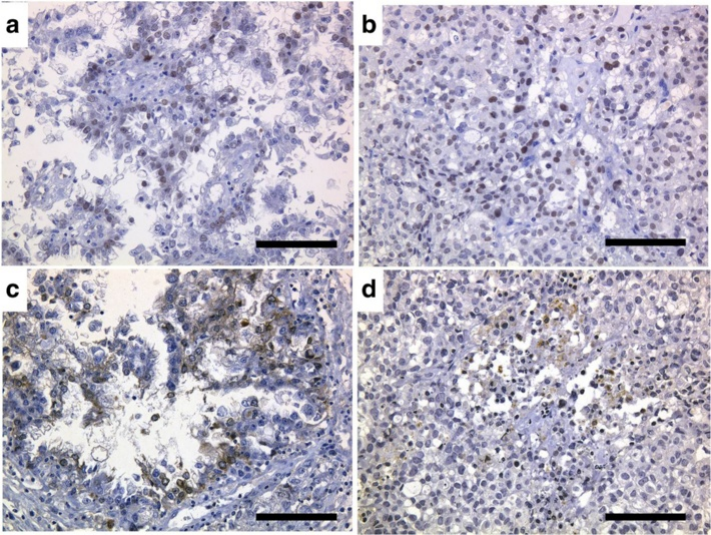
Immunohistochemical stainings of the original tumor (**a**, **c**) and heterotransplanted tumor (**b**, **d**) with HNF-1β (**a**, **b**) and Annexin IV (**c**, **d**). Cancer cells were positive for HNF-1β and Annexin IV (**a**, **b**, **c**, **d**; bar = 100 μm)

CA 125, CA 19–9, HNF-1β, and Annexin IV were demonstrated immunohistochemically in cancer cells from the original tumor and heterotransplanted tumors (Figs. 10 and 11). Estrogen receptor and progesterone receptor were not detected using immunocytochemistry.

### Chemosensitivity

The EC50 values of anti-cancer drugs for HCH-1 cells are listed in Table 1 (Additional file 2: Figure S1).

**Table 1:** Chemosensitivity using a 3-(4,5-dimethylthiazol-2-yl)-2,5-diphenyl tetrazolium bromide assay

Drug	EC50	PPC
	(μg/mL)	(μg/mL)
ACD	0.008	0.08
ADM	0.62	0.4
BLM	184.4	3.3
CBDCA	27.0	37.1
CDDP	3.9	8.5
CPA	6.9	4.3
CPT-11	0.511	0.05
5-FU	61.5	15.3
MMC	1.4	2.4
MTX	>300	3
PTX	68.6	11.8
VCR	61.7	0.1
VLB	18.6	0.15
VP-16	52.4	13

EC50, effective concentration for 50 % kill; PPC, peak plasma concentration taken intravenously; *ACD* actinomycin D, *ADM* doxorubicin, *BLM* bleomycin, *CBDCA* carboplatin, *CDDP* cisplatin, *CPA* 4-hydroperoxy-cyclophosphamide, *CPT-11* irinotecan hydrochloride, *5-FU* 5-fluorouracil, *MMC* mitomycin C, *MTX* methotrexate, *PTX* paclitaxel, *VCR* vincristine, *VLB* vinblastine, *VP-16* etoposide

### Mutational analysis

The amount of 8.1 μg DNA was extracted from 2×10^6^ cells. No variant was found in hotspot locations of 50 cancer genes but 12 of called variants outside of hotspot locations were found (Table 2).

**Table 2:** Variants outside of hotspot locations of 50 cancer genes

	Chromosome	Position	Ref Allele	Variant	Allele Type	Frequency	Variant Type	Allele Source^a^	Gene Symbol	Coverage	AA Ref	AA Variant
1	chr3	178917005	A	G	Heterozygous	40.2	SNP	Novel	PIK3CA	1950	unknown	unknown
2	chr4	1807894	G	A	Homozygous	100	SNP	Novel	FGFR3	1222	T	T
3	chr4	55141055	A	G	Homozygous	100	SNP	Novel	PDGFRA	1755	I	I
4	chr4	55972974	T	A	Homozygous	100	SNP	Novel	KDR	1987	Q	H
5	chr4	153249443	C	T	Homozygous	100	SNP	Novel	FBXW7	1998	V	V
6	chr5	112175770	G	A	Homozygous	100	SNP	Novel	APC	1981	A	A
7	chr5	149433596	TG	GA	Heterozygous	32.1	MNP	Novel	CSF1R	1993	unknown	unknown
8	chr7	55249063	G	A	Heterozygous	52.1	SNP	Novel	EGFR	1999	Q	Q
9	chr13	28610183	A	G	Homozygous	100	SNP	Novel	FLT3	1999	unknown	unknown
10	chr17	7579472	G	C	Heterozygous	49.2	SNP	Novel	TP53	1976	P	R
11	chr19	1220321	T	C	Heterozygous	55.4	SNP	Novel	STK11	1894	unknown	unknown
12	chr20	57484611	G	A	Heterozygous	48.9	SNP	Novel	GNAS	1994	R	H

*Ref* reference, *AA* amino Acid

^a^Allele Source, whether a variant is hotspot: Novel or Hotspot

## Discussion

Despite recent advances in cell culture it is still difficult to establish a cell line, chiefly because of contamination with fibroblasts. Fibroblasts usually grow faster than epithelial cells and cause them to float. As fibroblasts are detached faster than epithelial cells by trypsin, we removed every fibroblast and most of the epithelial cells leaving a few remaining epithelial colonies. These cells initially grew slowly, but eventually grew well, and the first subculture was made 2 months after the primary culture.

HCH-1 is a newly established cell line originating from clear cell carcinoma of the ovary. To obtain convincing evidence that HCH-1 is a strictly human ovarian clear cell carcinoma cell line, we investigated its biological characteristics and demonstrated the following:

1. The culture material was from a human clear cell carcinoma of the ovary which was positive for HNF-1β and Annexin IV.
2. HCH-1 has been kept viable in culture for over 230 months.
3. The cultured cells are neoplastic, show pleomorphism, and pile up easily without contact inhibition.
4. The chromosomes show a human karyotype and aneuploidy.
5. This line could be transplanted into SCID mice and formed tumors histologically similar to the original tumor.

Ovarian clear cell carcinoma cell lines have previously been used for basic research, but only eleven (HUOCA-II [16], RMG-I [17], OCC1 [18], RMG-II [19], OVISE [20], OVTOKO [20], JHOC-5 [21], JHOC-6 [21], SMOV-2 [22], TAYA [23], RMG-V [24], TU-OC-1 [25]) have been described in detail in the scientific literature (Table 3). Each cell line originated from a tumor, metastatic tumor, recurrent tumor, or ascites. The population doubling time varied greatly (24–96 h), the modal chromosome numbers were in the diploid range or more and all cells showed karyotypical abnormalities. HCH-1 is the only cell line in which the chromosome number is in the hypodiploid range. The hypodiploidy means the deletion of the chromosomes which are not necessarily important for cancer cells. It will thus provide an additional and useful model system for studying carcinogenesis.

**Table 3:** Cell lines from ovarian clear cell carcinoma

Cell line	Age	Materials	Chromosome number	DT (hour)	Transplantability	Immunotaining	Characteristics
HUOCA-II (1987)	51	Ovary	46 (mode)	24, 28	Yes	-	Tumor angiogenesis factor
RMG-I (1988)	34	Ascites	47 (mode)	60	Yes	BFP, ferritin, PLAP	-
OCC1 (1990)	47	Ascites	70–77	36, 38	Yes	-	Production of CA125
RMG-II (1991)	53	Ascites	hypertetraploid	58	No	CA125, TPA, MA602-1, MA602-6	Production of CA125, TPA, MA602-1, MA602-6
OVISE (1995)	40	Metastatic tumor	62 (59–65)	60	Yes	CA125, CA19-9, EGFR, ER(−), PgR(−)	Production of CA19-9, CA125, TPA
OVTOKO (1995)	78	Metastatic tumor	78 (76–83)	70	Yes	EGFR, ER(−), PgR(−)	-
JHOC-5 (1999)	47	Ovary	74–85	52	No	-	CA125
JHOC-6 (1999)	43	Recurrent tumor	46–49	70	Yes	-	CA125
SMOV-2 (1999)	46	Tumor	85–92	48.2	Yes	-	p53 mutation (−)
TAYA (2002)	43	Ascites	69–74	50	No	-	p53 Exon5 point mutation, PTEN mutation (−)
RMG-V (2005)	52	ascites	83 (77–85)	15.5	No	-	-
TU-OC-1 (2013)	65	Ovary	64–69	38.4	Yes	-	PIK3CA mutation (+)
HCH-1	67	Ovary	39–44	48.7, 66.4	Yes	CA125, CA19-9, ER(−), PgR(−)	production of CA19-9, CA125, TPA

*DT* doubling time (hour)

Five cell lines are reported to express tumor markers, but only two were positive immunohistologically. Tumor markers are useful not only in diagnosing the ovarian cancer but also in detecting tumor recurrence and assessing therapy. In our patient, CA125 and CA19-9 were useful tumor markers because of their high serum levels preoperatively. HCH-1 is the second reported cell line of ovarian clear cell carcinoma which produces both CA 125 and CA 19–9 markers. Therefore it will provide a useful, additional tool in studies of those ovarian cancers that produce tumor markers.

The ovary is a target organ for steroid hormones, but the cell lines OVISE, OVTOKO and HCH-1 do not possess estrogen or progesterone receptors as judged by immunohistochemical staining. Because the action, mechanism and localization of steroid hormone receptors are still relatively poorly understood, HCH-1 can be used as an additional negative control in hormone receptor studies.

Chemotherapy is an important adjunct to surgery in the treatment of ovarian cancer. The CAP (CPA, ADM, CDDP) regimen had, until recently, been used for ovarian epithelial carcinoma, but has now been largely superseded by a regimen of paclitaxel and carboplatin. CPT-11 has also been used for clear cell carcinoma and recurrent tumors [26]. However, these management strategies are not always effective.

The MTT assay is considered a rapid and accurate method of screening for drug responsiveness of cultured cells and so was selected for our chemosensitivity testing. In vitro sensitivity was defined as more than 50 % growth inhibition at peak plasma concentrations. According to this criterion, HCH-1 cells are sensitive to ACD, CBDCA, CDDP and MMC. These results do not, however, reflect our patient’s clinical course, suggesting that these drugs were metabolized before they could exert their effect. The MTT assay may provide a potentially useful approach for evaluating individual chemosensitivity profile but new principle for chemosensitivity testing, in vivo or genomic testing, would be expected.

Recently numerous genetic alterations revealed in ovarian cancer. The PIK3CA [3] gene was found to be specifically mutated in ovarian clear cell carcinoma. Then ARID1A [4] mutation and the consequent loss of expression are frequently observed. As a molecular study, somatic mutations were assessed using Panels of 50 most commonly reported oncogene and tumor suppressor genes. HCH-1 had no variant in hotspot locations of 50 genes including KRAS, PIK3CA, PTEN and TP53, but 12 of called variants outside of hotspot locations, those roles have not been known well. Important roles of carcinogenesis might be found in these variants or another cancer genes. Unfortunately, ARID1A was not included in this Panel.

## Conclusions

Since it is impossible to establish a cell line from the malignant tumor of each patient, the cell line that we established, characterized and report in this paper may be very useful in basic research on ovarian cancer, though the results of these analyses could not necessarily apply to new practical use. We have much to learn about the pathogenesis of clear cell carcinoma and this extra line of enquiry may lead us to a better understanding of how to treat and cure this serious disease.

## Abbreviations

5-FU, 5-fluorouracil; ACD, Actinomycin D; ADM, Doxorubicin; AFP, α-feto-protein; BLM, Bleomycin; CBDCA, Carboplatin; CDDP, Cisplatin; CEA, Carcinoembryonic antigen; CPA, Cyclophosphamide; CPT-11, Irinotecan SN-38; DAB, Diaminobenzidine; DMSO, Dimethyl sulfoxide; EC50, Effective concentration for 50 % kill; EDTA, Ethylenediamine-tetraacetic acid; HCG, Human chorionic gonadotropin; HE, Hematoxylin-eosin; ISCN, International System for Human Cytogenetic Nomenclature; MMC, Mitomycin C; MTT, 3-(4,5-dimethyl-2-thiazolyl)-2,5-diphenyl-2H tetrazolium bromide; MTX, Methotrexate; PBS, Phosphate buffered saline; PTX, Paclitaxel; SCC, Squamous cell carcinoma; TPA, Tissue polypeptide antigen; VCR, Vincristine; VLB, Vinblastine; VP-16, Etoposide.

## Additional files

Additional file 1: Table S1. List of the 50 genes targeted by the Ion AmpliSeq™ Cancer Hotspot Panel v2 (Ion Torrent). (XLS 31 kb) Additional file 2: Figure S1. Chemosensitivity of HCH-1 cells. HCH-1 cells are considered sensitive to ACD, CBDCA, CDDP and MMC. (JPG 76 kb)
