# Supplementary figures and images for: Establishment and characterization of a cell line (HCH-1) originating from a human clear cell carcinoma of the ovary
Source: J Ovarian Res. 2016 Jun 4;9:32. doi: 10.1186/s13048-016-0242-y (PMC4893251; doi:10.1186/s13048-016-0242-y)

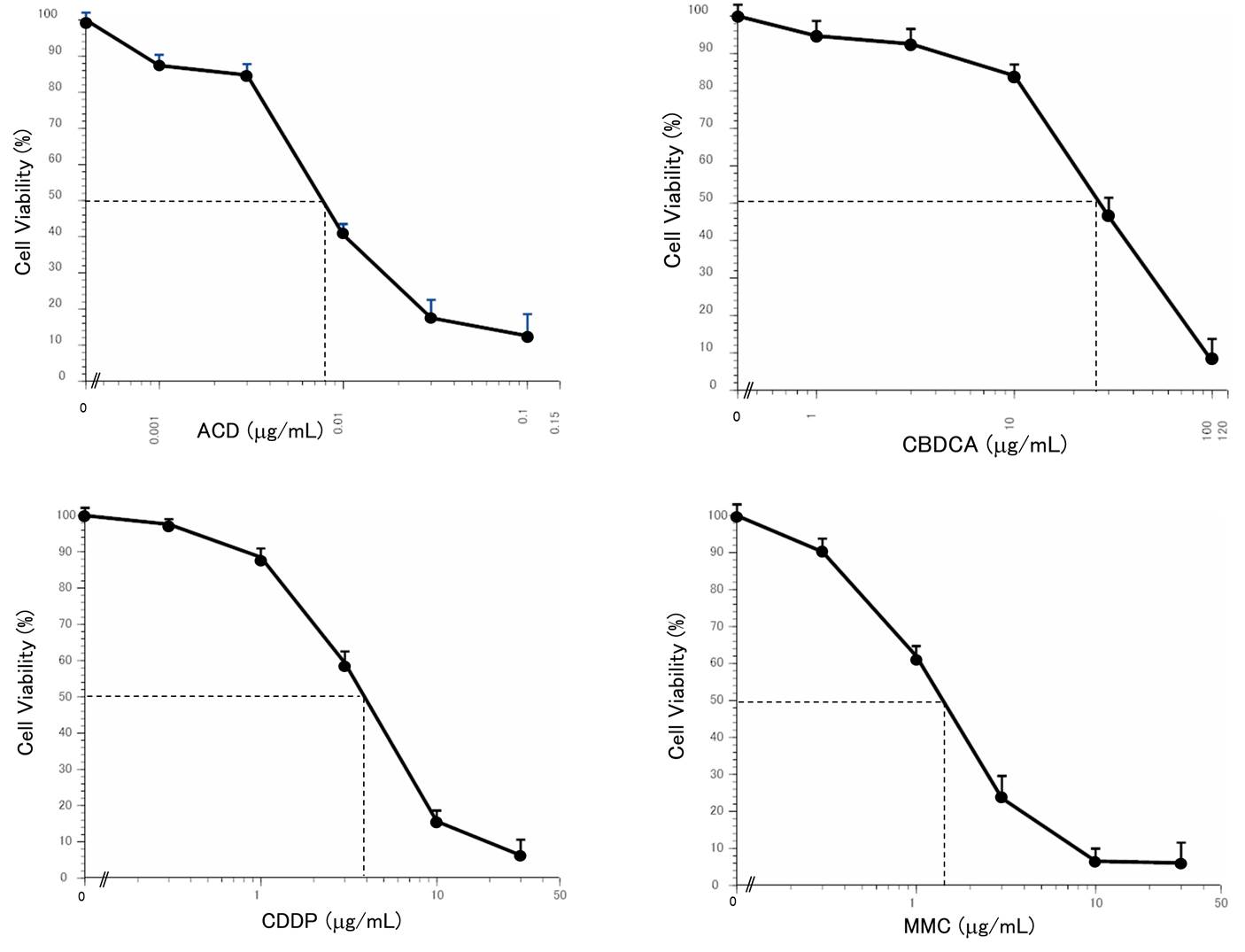

Supplement: Additional file 2: Figure S1. — Chemosensitivity of HCH-1 cells. HCH-1 cells are considered sensitive to ACD, CBDCA, CDDP and MMC. (JPG 76 kb) [file 13048_2016_242_MOESM2_ESM.jpg]
